# *Aspergillus* march: from ABPA to aspergilloma to subacute invasive aspergillosis

**DOI:** 10.1186/s13223-016-0170-9

**Published:** 2016-12-03

**Authors:** Vikas Dogra, Ankit Kumar Sinha, Rajat Saxena, Deepak Talwar

**Affiliations:** 1 Rajiv Gandhi Superspeciality Hospital, Tahirpur, Delhi, 110093 India; 2 Metro Center for Respiratory Diseases, Metro Multispeciality Hospital, Sector 11, Noida, Uttar Pradesh India

**Keywords:** Asthma, *Aspergillus*, Aspergilloma

## Abstract

**Background:**

*Aspergillus* is a ubiquitous fungus responsible for allergic as well as saprophytic and invasive manifestations depending on host’s immune status. The following case report demonstrates progression of allergic manifestations of *Aspergillus* to its invasive form in an individual with decreasing immunity. This can lead to uncertainties in diagnosis and management.

**Case presentation:**

A 28-year-old male, non smoker, known case of ABPA (allergic bronchopulmonary aspergillosis) was admitted with complaints of cough for 1 month, associated with recurrent episodes of hemoptysis for last 5 days. CT Thorax revealed homogenous dense round opacity in right upper lobe which replaced previous fibrocalcific bronchiectatic lesion with cavity and aspergilloma, bulging across the major fissure with fibrotic strands extending to periphery in all directions. Post-pneumonectomy microscopic examination revealed *Aspergillus* hyphae invading blood vessels.

**Conclusion:**

There is a need for close clinical and radiologic follow up of patients with *Aspergillus* and our patient demonstrated overlap of complete spectrum of *Aspergillus* disease with march from one end to the other end.

## Background

*Aspergillus* is a fungus with ubiquitous presence. It is responsible for spectrum of diseases depending upon the host immune status varying from allergic bronchopulmonary aspergillosis (ABPA) in atopics, aspergilloma in chronic lung cavity, and chronic pulmonary aspergillosis (CPA) or invasive pulmonary aspergillosis (IPA) in immunocompromized. However, overlap among them has been observed as immune status changes because of treatment with steroids for ABPA or development of diabetes which itself leads to immunosuppression. We present a rare case with ABPA which progressed to develop aspergilloma and finally to subacute invasive aspergillosis over a period of 10 years.

## Case presentation

**Fig. 1:**
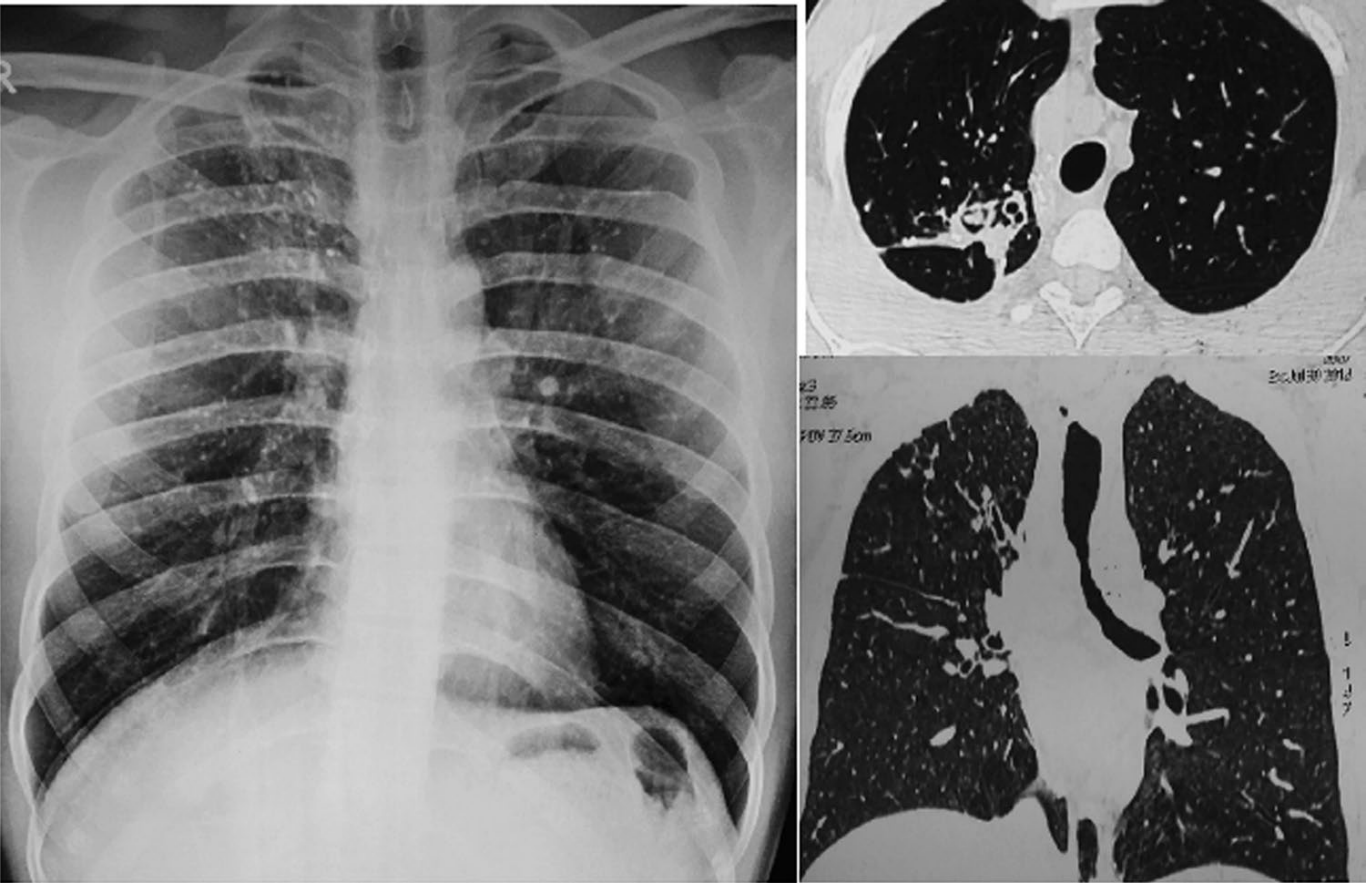
CXR showing *right upper* lobe opacity which on HRCT showed central bronchiectasis in *right upper* lobe anterior segment

**Fig. 2:**
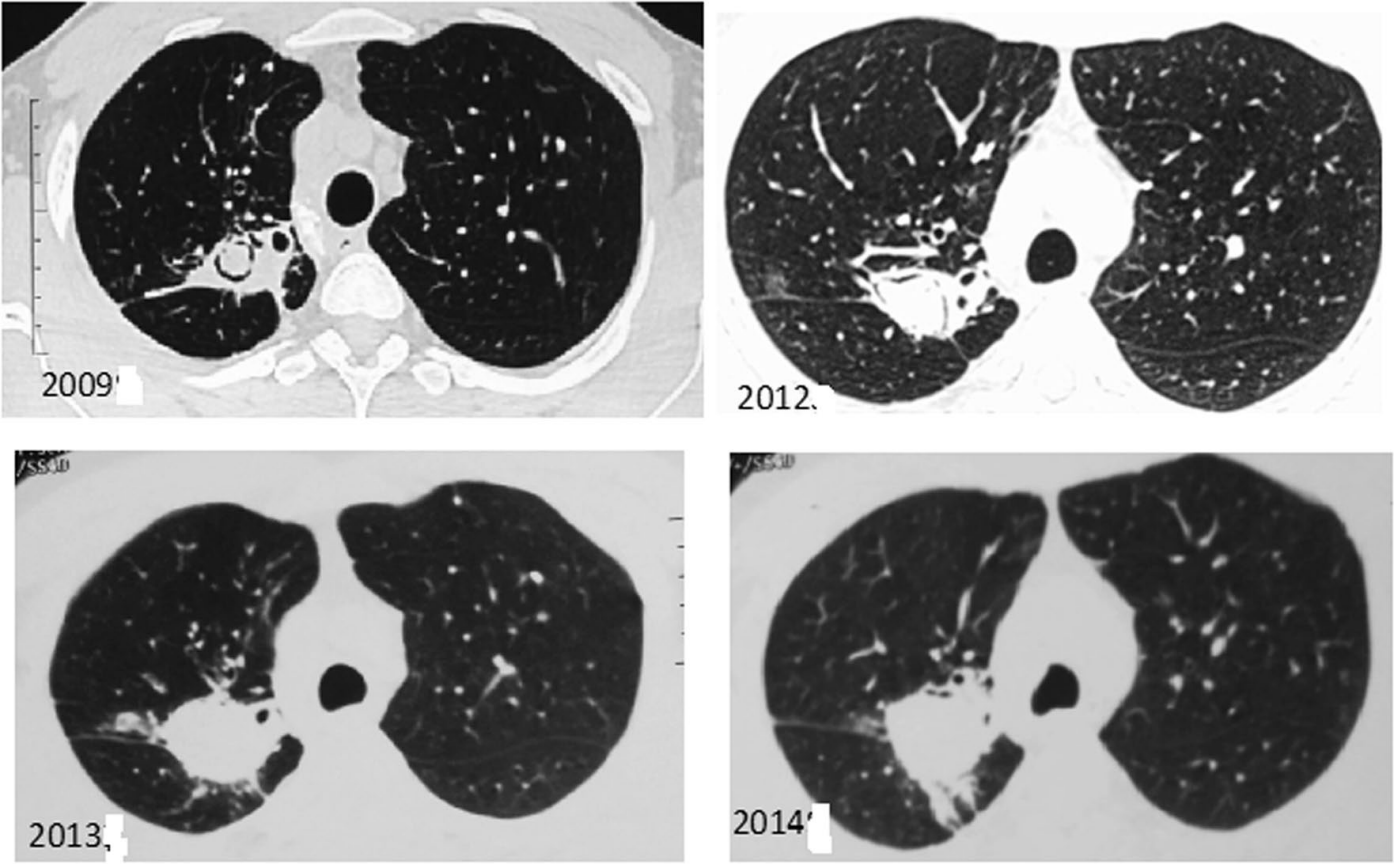
HRCT cuts from 2009 to 2014 showing development of aspergilloma in *right upper* lobe which grew over 5 years

A 28-year-old male, non smoker, known case of ABPA was admitted with complaints of cough with scant expectoration for 1 month, associated with recurrent episodes of hemoptysis for last 5 days. He had been diagnosed as ABPA 10 years back with Skin Prick Test (SPT) strongly positive for *Aspergillus fumigatus, flavus* and *niger*. Total Serum IgE was 1200 KU/L with specific IgE and IgG positive against *A. fumigatus*. He was on treatment with steroids for the same. 5 years ago he was admitted with mild hemoptysis, fever and breathlessness for 2 weeks. Contrast enhanced CT chest showed mucoid impaction (HAM—High attenuation mucus) and central bronchiectasis in right upper lobe (Fig. 1). He received Itraconazole for 4 months. Subsequently, he had repeated admissions for 3 consecutive years with similar complaints and repeat CT done revealed fibrocalcific lesion in apical and posterior segments of right lung upper lobe with a cavity showing soft tissue attenuation and air crescent sign suggestive of mycetoma (Fig. 2). He was advised surgery, which was deferred by patient and he took Voriconazole for 6 weeks and later itraconazole for 6 months from elsewhere and was lost to follow up. He had been on oral steroids for 10 years receiving on an average 6 months of steroids (Prednisolone starting from 60 mg then tapered). For last 2 years he was on oral Deflazacort 12 mg with doses increased during exacerbations.

**Fig. 3:**
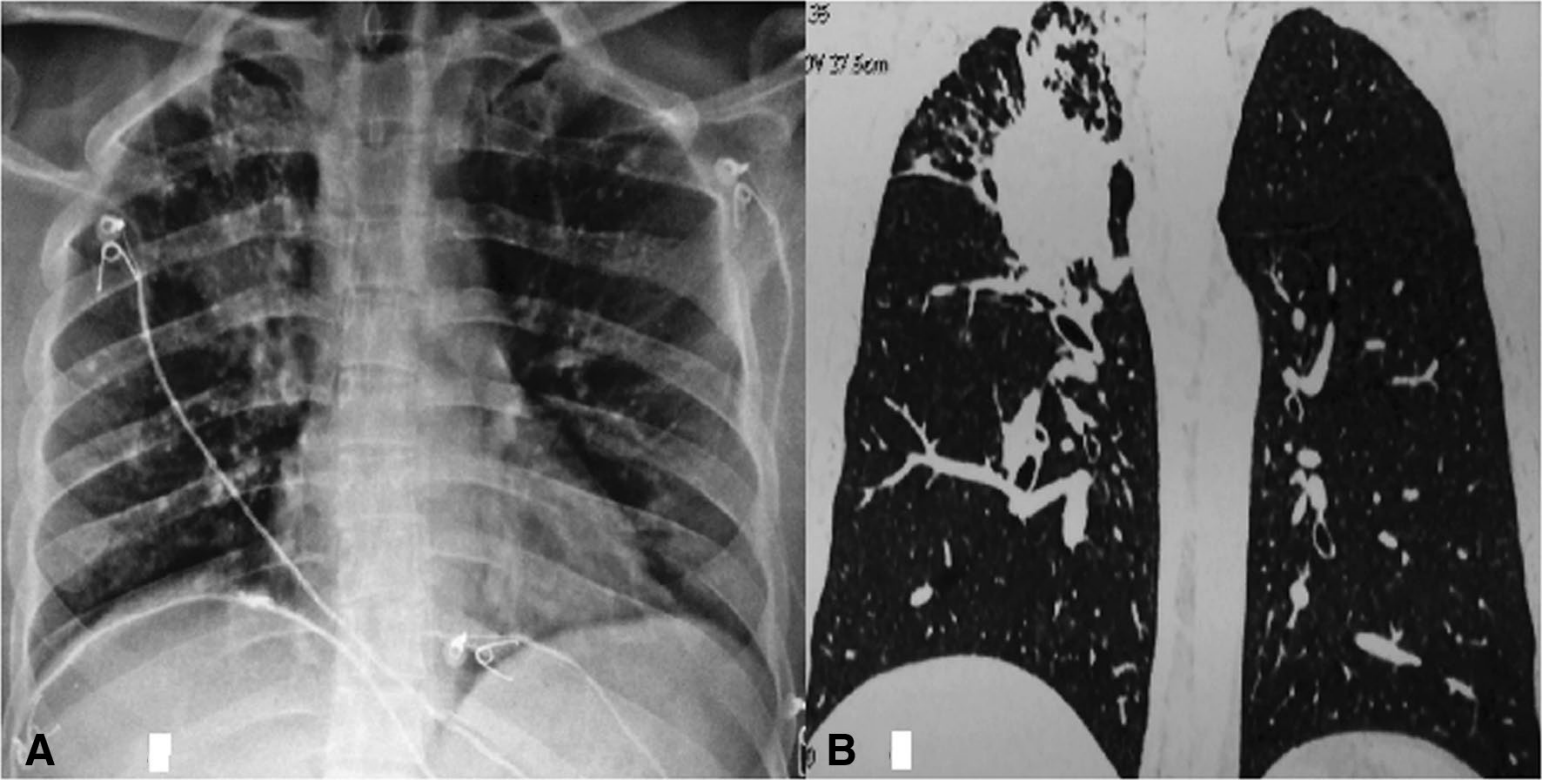
**A** Chest Xray showing opacity *right upper* lobe **B** HRCT sagittal cut showing expanded aspergilloma pushing across oblique fissure with peripheral infiltrates

At present admission his resting peripheral capillary oxygen saturation was 89% on room air. Chest X ray revealed hyperinflated lung fields with focal homogenous opacity with infiltrates in right upper zone (Fig. 3). CT Thorax revealed homogenous dense round opacity in right upper lobe which replaced previous fibrocalcific bronchiectatic lesion with cavity and aspergilloma, bulging across the major fissure with fibrotic strands extending to periphery in all directions. Sputum smears and culture examinations for mycobacteria, and fungi were negative.

**Fig. 4:**
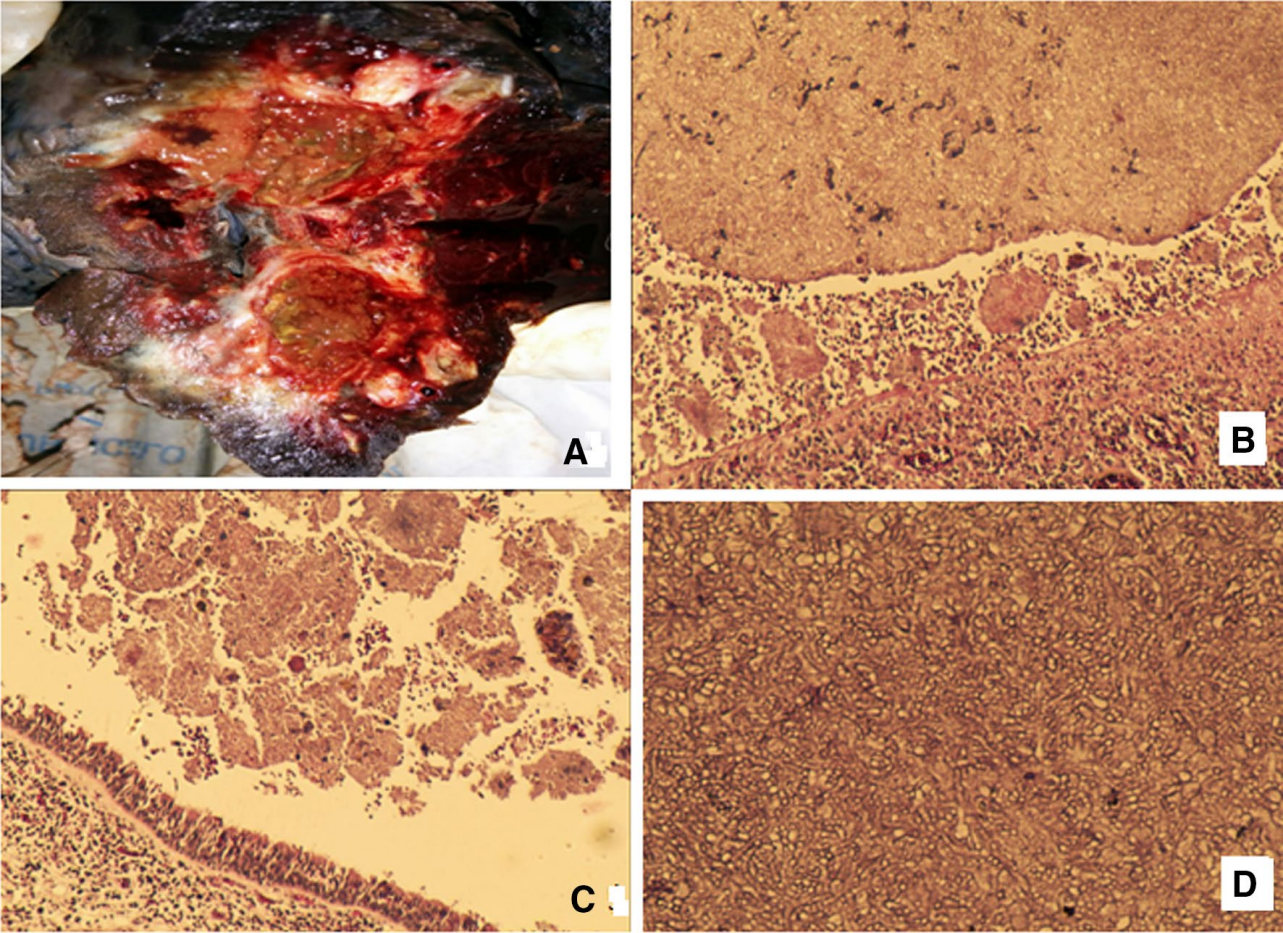
**A** Gross right pneumonectomy specimen with large fungus ball in cavity with fibrosis. **B** Lung parenchyma blood vessel showing infiltration with fungal hyphae). **C** Intra bronchial fungal elements. **D** fungal ball showing acutely branching septate hyphae of *Aspergillus*

In view of recurrent hemoptysis, unilateral disease and worsening radiological disease with acceptable lung functions, patient underwent thoracotomy. The lesion was found to be extending across major fissure with involvement of right lower lobe apical segment and decision for pneumonectomy was taken. Gross examination of resected lung showed cavity in the upper lobe filled with solid debris (Fig. 4A) which on microscopic examination shows fungal colonies comprising of acutely branching septate fungal hyphae with invasion of surrounding bronchi, alveolar tissues and blood vessels. Surrounding parenchyma showed emphysematous changes with septate branching fungal hyphae and minimal fibrosis of the cavity wall and surrounding pleural consistent with the diagnosis of complex mycetoma with locally invasive aspergillosis (Fig. 4B–D). Few central bronchiectatic segments were seen in the upper lobe.

Patient had uneventful recovery and is doing well. His serum galactomannan was 2.5 and given oral voriconazole for 6 weeks and repeat serum galactomannan was negative and now patient is being regularly followed in OPD.

## Discussion

*Aspergillus* causes a spectrum of diseases depending upon the immune status of the host (Table 1) [1].

**Table 1:** Manifestations of pulmonary aspergillosis

*Simple colonization*	
*Allergic bronchopulmonary aspergillosis (ABPA)*	
*Chronic pulmonary aspergillosis*	
Simple aspergilloma	
Chronic cavitary pulmonary aspergillosis (CCPA)	
Chronic fibrosing pulmonary aspergillosis	
Aspergillus nodule	
*Subacute invasive aspergillosis (SAIA)*	
*Angioinvasive aspergillosis*	
*Airway invasive aspergillosis*	

Both ABPA and aspergilloma are its noninvasive forms. Aspergilloma is a fungus ball that develops in a pre-existing cavity within the lung parenchyma, while ABPA is a hypersensitivity manifestation in the lungs that almost always affects patients with asthma or cystic fibrosis. Tissue necrosis and invasion of blood vessels does not occur. Aspergillomas presenting with recurrent hemoptysis are treated by surgery. Our case also highlights failure of antifungals in management of aspergillomas as patient tried though for limited period to avoid surgery.

Subacute invasive pulmonary aspergillosis (SAIA) or Chronic Necrotizing Aspergillosis is a locally invasive disease manifesting as a syndrome of slowly progressive cavitary lung disease, chronic respiratory symptoms, and the presence of precipitating antibodies to *Aspergillus* [2]. It typically occurs in patients with a depressed immune system, but not as profoundly immunocompromised [2]. It is commonly observed in diabetics, patients on corticosteroids or with underlying lung disease like COPD. SAIA usually runs a slowly progressive course over weeks to months. It is the finding of tissue invasion that allows this entity to be distinguished from the more common aspergilloma.

Invasive pulmonary aspergillosis (IPA) is a severe disease, found in immunocompromised patients. Certain factors have been documented to predispose an individual to IPA (Table 2) [3.

**Table 2:** Classical risk factors for invasive pulmonary aspergillosis

Prolonged neutropenia (<500 cells/mm3 for >10 days)	
Transplantation (highest risk is with lung transplantation and HSCT)	
Prolonged (>3 weeks) and high-dose corticosteroid therapy	
Hematological malignancy (risk is higher with leukemia)	
Chemotherapy	
Advanced AIDS	
Chronic granulomatous disease	

There are reports documenting IPA in immunocompetent patients who do not have the clearly known risk factors especially severe COPD and critically ill patients [4].

Aspergilloma is an unusual complication of ABPA. Cavitation is known to occur in ABPA but still aspergilloma formation is not common. There have been case reports documenting aspergilloma and ABPA in patients with difficult to control asthma [5]. A case series of 179 patients of ABPA out of which eight also had aspergilloma concluded that concurrent presentation of ABPA and aspergilloma was associated with an immunologically severe disease with higher IgE levels and more extensive bronchiectasis and risk of recurrent relapses [6]. Spontaneous disappearance of aspergilloma has been reported in 5% of cases while they rarely have been reported to increase in size [7]. Thickening of cavity wall and adjacent pleura has been demonstrated to be due to hypersensitivity reaction secondary to fungus as its reversible with resolution of aspergilloma. A chronic mycetoma may suddenly breakdown and become a rapidly invasive pulmonary infection.

A case report by Shah et al. found ABPA, AAS (allergic *Aspergillus* sinusitis), and aspergilloma occurring simultaneously in the same patient [8].

In present case development of ‘Simple Aspergilloma’ in ABPA was observed at follow up, which kept on increasing in size with destruction of surrounding lung parenchyma and pleural thickening converting it into a ‘Complex Aspergilloma’. It eventually became invasive in the same patient. Possible reason might have been immunocompromized due to corticosteroid therapy, which he received for management of ABPA. This presents a unique therapeutic challenge, as corticosteroids are the mainstay for management of ABPA.

Subacute invasive aspergillosis presents clinically with non specific symptoms with mild to moderate hemoptysis. Radiologically dense circumscribed lesion with or without halo sign is seen. Positive Aspergillus on microscopy from biopsy specimen and positive serum antigen test for Aspergillus are also observed. All these features have been demonstrated in our case. In such cases surgical resection is indicated if significant hemoptysis occurs. In addition to serving as a diagnostic tool, serial galactomannan determinations have been suggested to be useful for monitoring the treatment response or as a surrogate endpoint for outcomes of invasive aspergillosis.

## Conclusion

Our patient demonstrated overlap of complete spectrum of Aspergillus disease with march from one end to the other end. This case emphasizes importance of regular clinical and radiologic follow up of patients with ABPA.

## Abbreviations

abpa: allergic bronchopulmonary aspergillosis
cpa: chronic pulmonary aspergillosis
ipa: invasive pulmonary aspergillosis
spt: skin prick test
ham: hyper attenuated mucus
ccpa: chronic cavitary pulmonary aspergillosis
saia: subacute invasive aspergillosis
copd: chronic obstructive pulmonary disease
aas: allergic Aspergillus sinusitis

## References

[1] Denning DW, Cadranel J, Beigelman C (2016). Chronic pulmonary aspergillosis: rationale and clinical guidelines for diagnosis and management. Eur Respir J.

[2] Saraceno JL, Phelps DT, Ferro TJ (1997). Chronic necrotizing pulmonary aspergillosis: approach to management. Chest.

[3] Kousha M, Tadi R, Soubani AO (2011). Pulmonary aspergillosis: a clinical review. Eur Respir Rev.

[4] Thommi G, Bell G, Liu J, Nugent K (1991). Spectrum of invasive pulmonary aspergillosis in immunocompetent patients with chronic obstructive pulmonary disease. South Med J.

[5] Shah A (2010). Concurrent allergic bronchopulmonary aspergillosis and aspergilloma: is it a more severe form of the disease?. Eur Respir Rev.

[6] Agarwal R, Aggarwal AN, Garg M, Saikia B, Gupta D, Chakrabarty A (2012). Allergic bronchopulmonary aspergillosis with aspergilloma: an immunologically severe disease with poor outcome. Mycopathologia.

[7] Pratap H, Dewan RK, Singh L, Gill S, Avaddadi S (2007). Surgical treatment of pulmonary aspergilloma: a series of 72 cases. Indian J Chest Dis Allied Sci.

[8] Shah A, Panjabi C (2006). Contemporaneous occurrence of allergic bronchopulmonary aspergillosis, allergic Aspergillus sinusitis, and aspergilloma. Ann Allergy Asthma Immunol.

